# A La Autoantigen Homologue Is Required for the Internal Ribosome Entry Site Mediated Translation of Giardiavirus

**DOI:** 10.1371/journal.pone.0018263

**Published:** 2011-03-29

**Authors:** Srinivas Garlapati, Ashesh A. Saraiya, Ching C. Wang

**Affiliations:** Department of Pharmaceutical Chemistry, University of California San Francisco, San Francisco, California, United States of America; Emory University, United States of America

## Abstract

Translation of Giardiavirus (GLV) mRNA is initiated at an internal ribosome entry site (IRES) in the viral transcript. The IRES localizes to a downstream portion of 5′ untranslated region (UTR) and a part of the early downstream coding region of the transcript. Recent studies indicated that the IRES does not require a pre-initiation complex to initiate translation but may directly recruit the small ribosome subunit with the help of a number of *trans*-activating protein factors. A La autoantigen homologue in the viral host *Giardia lamblia*, GlLa, was proposed as one of the potential *trans*-activating factors based on its specific binding to GLV-IRES *in vitro*. In this study, we further elucidated the functional role of GlLa in GLV-IRES mediated translation in *Giardia* by knocking down GlLa with antisense morpholino oligo, which resulted in a reduction of GLV-IRES activity by 40%. An over-expression of GlLa in *Giardia* moderately stimulated GLV-IRES activity by 20%. A yeast inhibitory RNA (IRNA), known to bind mammalian and yeast La autoantigen and inhibit Poliovirus and Hepatitis C virus IRES activities *in vitro* and *in vivo*, was also found to bind to GlLa protein *in vitro* and inhibited GLV-IRES function *in vivo*. The C-terminal domain of La autoantigen interferes with the dimerization of La and inhibits its function. An over-expression of the C-terminal domain (200–348aa) of GlLa in *Giardia* showed a dominant-negative effect on GLV-IRES activity, suggesting a potential inhibition of GlLa dimerization. HA tagged GlLa protein was detected mainly in the cytoplasm of *Giardia*, thus supporting a primary role of GlLa in translation initiation in Giardiavirus.

## Introduction

Recognition of the initiation codon by small ribosomal subunit is a key step in translation initiation. In eukaryotes, cap-dependent translation is initiated by the binding of a pre-initiation complex (the 40S ribosomal subunit combined with eIF1, eIF1A, eIF3 and eIF2-GTP-tRNA) to the 5′ cap of mRNA through an interaction with eIF4G in the eIF4F complex bound to the cap. This complex then initiates a downstream scanning along the mRNA for the initiation codon to begin translation [1]. In an alternative mechanism, direct binding of a pre-initiation complex (the naked 40S ribosome plus a few protein factors) to the initiation codon is made possible by highly structured mRNA sequences known as internal ribosome entry sites (IRESs) [2]–[4]. IRESs were initially identified in the 5′ untranslated regions (UTRs) of uncapped messages of picornaviruses [5]. Subsequently, IRESs were also identified among members of flaviviruses and dicistroviruses [2]. Recently, numerous capped cellular mRNAs were discovered also to contain IRESs in their 5′-UTRs and shown to utilize IRES mediated translation initiation when normal cap dependent translation is severely compromised during conditions of cell stress, cell cycle, development and diseases [3].

Most IRESs require only a subset of canonical initiation factors, whereas others do not require any additional factors to initiate translation [6]. Some also require a set of non-canonical initiation factors known as IRES *trans*-activating factors (ITAFs) [4], [6]. Distinctive sets of ITAFs have been indentified with specific types of viral or cellular IRESs [6], [7]. La autoantigen was the first ITAF identified that stimulated Poliovirus (PV) IRES function both *in vitro* and *in vivo* [8], [9]. It was also demonstrated to bind to the Hepatitis C virus (HCV) IRES near the initiation codon and stimulate its activity in rabbit reticulocyte lysate [10]. The functional role of La protein in both viral IRESs was further confirmed by the inhibitory effects of reducing La protein by siRNA or by the lack of IRES function in a La dominant negative mutant, in which a C-terminal domain of La interferes with La protein dimerization [11]. La also stimulates the IRES activities of Encephalomyocarditis virus (EMCV) by alleviating the inhibitory effects of excess polypyrimidine tract binding protein (PTB) in the cell lysate [12]. For the cellular IRES function, La plays a critical role in the IRES mediated translation of X-linked inhibitor of apoptosis [13] and Bip mRNAs [14]. The La protein thus appears universally involved in regulating the functions of a variety of IRESs.

The role of La protein in regulating PV and HCV IRES function was further elucidated by the identification in *Saccharomyces cerevisiae* of a 60 nt RNA, which has a short hairpin structure and sequesters La and other RNA binding proteins in yeast cell [15]. It was referred to as the inhibitory RNA (IRNA) because it competes with Poliovirus and Hepatitis C virus IRESs for binding to La protein and inhibits their activity *in vitro* and *in vivo* [15]–[17]. This inherent property of IRNA to bind La protein was attributed to its specific secondary structure and not due to its primary sequence [15].

Giardiavirus (GLV) is a double stranded RNA virus that belongs to the *Totiviridiae* family [18] and specifically infects the vegetative cells (trophozoites) of the primitive eukaryote *Giardia lamblia*, which is a tetraploid [18]. Unlike picornaviruses or flaviviruses, Giardiavirus does not lyse nor retard the growth of its host *G. lamblia* [18], [19]. The 6,277 nt Giardiaviral transcript lacks a 5′ cap structure and its translation is initiated at an IRES [20]. The IRES encompasses both a downstream portion of 5′UTR and an early segment of the open reading frame [20]. Several secondary and tertiary structures of the IRES had been identified. Their potential roles in IRES function were extensively verified by expressing transcripts of various GLV- IRES mutants in bicistronic constructs in *Giardia* trophozoites [21]–[23]. The resistance of GLV-IRES function to the translation initiation inhibitor edeine *in vivo* indicated that it does not require recruitment of a pre-initiation complex for initiating translation [24]. The IRES was also found incapable of binding directly to the purified small ribosomal subunit from *Giardia*, suggesting the involvement of certain ITAFs in GLV IRES initiated translation [24]. Three putative ITAFs have since been identified by biochemical and bioinformatics approaches. A member of the helicase family IBP1 and two homologues of known viral IRES binding proteins SRp20 and La autoantigen (GlLa) were found to exhibit specific bindings to GLV IRES RNA *in vitro*, indicating potential involvement in the GLV IRES mediated translation [24]. In the current study, we further investigated the functional role of GlLa in GLV IRES mediated translation in *Giardia*.

## Results

### Knocking down GlLa Inhibits GLV IRES Function

**Figure 1:**
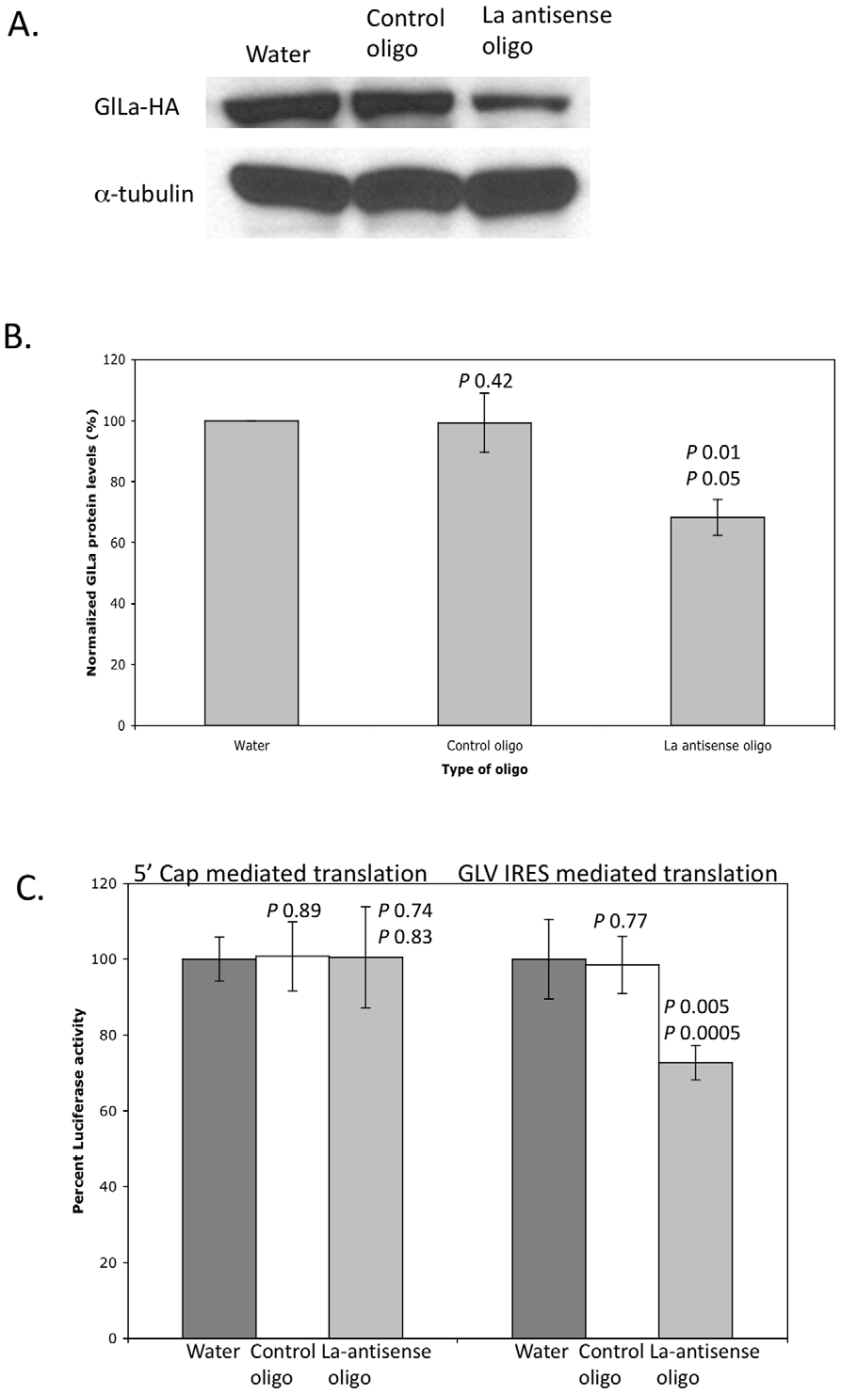
Effect of a reduced GlLa protein level on GLV-IRES activity. A) Western blot analysis at 48 hrs after the *Giardia* cells were transfected with water, control morpholino oligos, or La antisense morpholino oligo. 3XHA-GlLa, expressed under endogenous control, was stained with the anti-HA antibody whereas anti-α-tubulin antibodies stained tubulins served as loading controls. B) Relative levels of 3XHA-GlLa in transfected *Giardia* cells, calculated from densitometric scanning of Western blots from three independent transfection experiments (± S.D). C) Rluc activities expressed from 5′ cap-dependent and GLV-IRES mediated translations in 48 hrs post-transfected *Giardia* cells as described in A) (± S.D). Student t-test was conducted to calculate *P* values. *P* values above 0.05 are considered statistically insignificant, <0.05 significant, <0.01 very significant and <0.005 highly significant. The *P* values are indicated above each bar for control oligo (compared with water control) and antisense-GlLa oligo (top, compared with water control; bottom, compared with control oligo).

To determine if GlLa plays an essential role in GLV-IRES mediated translation in *Giardia*, the endogenous GlLa protein level was reduced by a custom synthesized antisense morpholino oligo [25]. The latter was introduced by electroporation into *Giardia* trophozoites (see Materials and Methods), in which one of the four chromosomal copies of the GlLa gene was tagged with a 3XHA epitope and expressed at the endogenous level [26]–[28]. Western analysis of the lysate from the electroporated cells with anti-HA antibodies indicated that after 48 hrs post-transfection, the GlLa protein level was reduced by 40% as compared to mock transfected cells or cells transfected with nonspecific oligos (Fig. 1A and B). Though this reduced expression was observed on the tagged GlLa, which constituted only one fourth of the total GlLa protein, we believe that the data provided an accurate estimate of reduction of the total GlLa protein.

The translation machinery in *Giardia* has been found to lack the mechanism of ribosome scanning [29]. Thus the GLV-IRES initiated translation of transcripts from uncapped monocistronic constructs resulted always in the same outcome from that of dicistronic constructs [23]. The 48-hr knockdown cells from above were then transfected with an uncapped *in vitro* transcript from the monocistronic template pC631Rluc containing a *Renilla* luciferase reporter driven by GLV-IRES. The Rluc activity was assayed 5 hrs post-transfection and the outcome showed that the GLV-IRES mediated translation of Rluc reporter was inhibited by ∼40% in the GlLa knockdown cells when compared to the controls (Fig. 1C). In contrast, cap-dependent translation of the same reporter gene under the same experimental conditions was not affected by GlLa knockdown, indicating that GlLa does not play a role in cap-mediated translation in *Giardia* cells (Fig. 1C).

### GlLa Stimulates GLV-IRES Function

**Figure 2:**
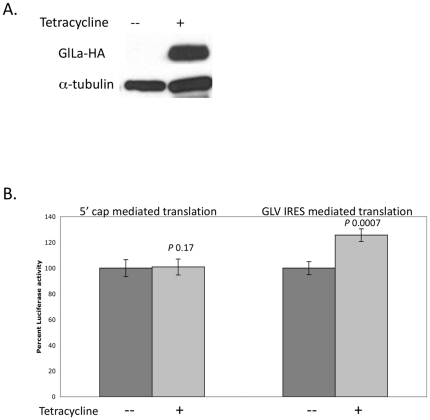
Effect of an over-expressed GlLa protein on GLV-IRES activity. A) Western analysis of GlLa-HA levels in un-induced and tetracycline-induced *Giardia* cells 3 hrs after transfection. GlLa-HA was detected by anti-HA antibody. The lower panel shows α-tubulin as the loading control. B) Relative Rluc expression from 5′-cap mediated or GLV-IRES mediated translation initiation in un-induced (black columns) or tetracycline-induced (grey coulmns) *Giardia* trophozoites 3 hrs after the previously described transfection. The results were derived from three independent transfection experiments (± S.D). *P* values are indicated above each bar in the graph.

HA (hemmaglutinin) tagged GlLa was expressed in *Giardia* using a tetracycline inducible Ran promoter system in a plasmid construct [30]. Western analysis of the lysate from transfected cells induced with tetracycline for 24 hrs indicated the presence of a HA tagged GlLa protein, whereas none were detected in the un-induced cells (Fig. 2A). To determine if the increased level of tagged GlLa has an effect on GLV-IRES function, the cells over-expressing HA-tagged GlLa was further transfected with an *in vitro* pC631Rluc transcript and assayed for Rluc activity 5–7 hrs post-transfection. The Rluc activity in Tet-induced cells was 25% higher than that in the un-induced cells, indicating that a higher level of GlLa protein has a stimulatory effect on GLV-IRES mediated translation (Fig. 2B). The cap-dependent translation of Rluc reporter was not changed in Tet-induced versus un-induced cells, indicating that GlLa exerts no effect on 5′ cap dependent translation initiation (Fig. 2B).

### IRNA binds GlLa *in vitro* and Inhibits GLV IRES Function *in vivo*

**Figure 3:**
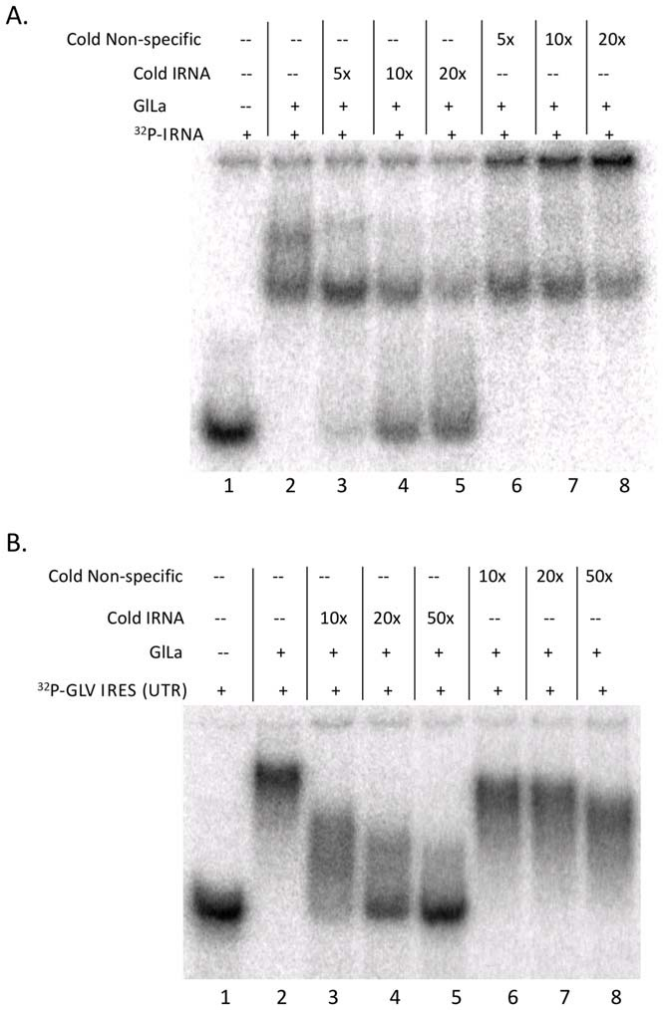
Yeast IRNA binds to GlLa and competes with the 5′ UTR portion of GLV-IRES RNA for binding. A) Binding of yeast IRNA to GlLa (lane 2) was competitively inhibited by 5 to 20 fold excess of cold yeast IRNA (lanes 3–5) but not by 5 to 20 fold non-specific cold 60 nt RNA (lanes 6–8). B) Binding of radiolabeled 5′ UTR of GLV-IRES RNA (lane 2) was competitively inhibited by 10 to 50-fold excess of cold yeast IRNA (lanes 3–5), but not by 10 to 50-fold excess of cold 60 nt non-specific RNA (lanes 6–8).

**Figure 4:**
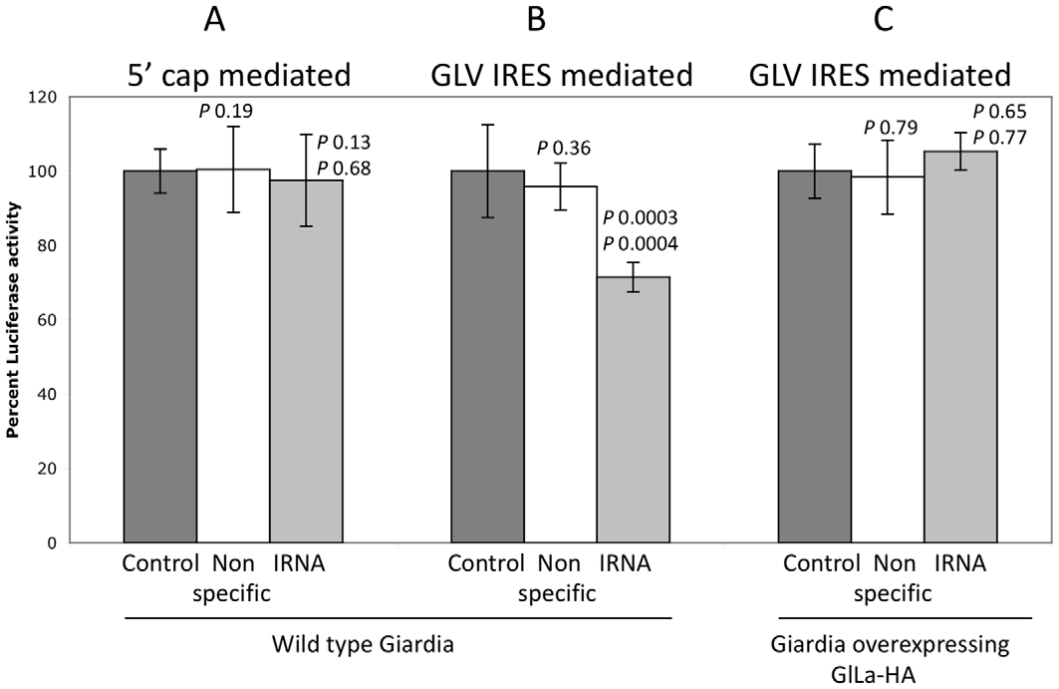
Yeast IRNA inhibits GLV-IRES activity *in vivo*. Relative Rluc activities in *Giardia* WB strain trophozoites transfected with Rluc reporter transcripts driven by 5′-cap (A) or GLV-IRES (B) in control (black), presence of the 60 nt non-specific RNA (white) or presence of yeast IRNA (grey). (C) Relative Rluc activities in *Giardia* WB cells over-expressing GlLa-HA and transfected with GLV-IRES driven RLuc reporter transcripts in the control (black), the presence of 60 nt non-specific RNA (white) or the presence of yeast IRNA (grey). The results were derived from three independent transfection experiments (± S.D). The *P* values are indicated above each bar for non-specific RNA (compared with control) and IRNA (top, compared with control; bottom, compared with non-specific RNA).

To further confirm the essential role of GlLa in mediating GLV IRES function, we used *in vitro* synthesized yeast IRNA, which has been shown to inhibit the functions of HCV-IRES and PV-IRES both *in vitro* and *in vivo* by specifically sequestering La protein [16], [17]. In a gel-shift assay, purified recombinant GlLa protein was shown to bind radiolabeled IRNA and the binding was competed out by 5 to 20 fold molar excess of unlabeled IRNA but not by an excess of an unlabeled non-specific RNA of a similar size (Fig. 3A). We then tested the ability of IRNA to compete with ^32^P-GLV-IRES for binding to GlLa protein in a gel-shift assay. Synthetic IRNA was found capable of replacing the radiolabeled 5′UTR portion of GLV IRES from binding to recombinant GlLa protein in a dose dependent manner, whereas a non-specific RNA of similar size did not exert any detectable effect (Fig. 3B). IRNA could thus block the binding between GlLa and GLV-IRES *in vitro*. To test if the same will happen *in vivo*, an excessive amount of IRNA was introduced with the transcript from pC631Rluc into *Giardia* cells via transfection and the Rluc activity was assayed after 5 hrs. The results indicated that GLV IRES mediated translation was reduced by 30% compared to mock transfected or non-specific RNA transfected cells (Fig. 4). The same experiment performed on cap-dependent translation of the reporter gene indicated that IRNA has no detectable effect.

To further confirm that the inhibitory effect of IRNA is due to a specific sequestering of GlLa protein, the *Giardia* trophozoites over-expressing GlLa (Fig. 2A) were transfected with IRNA and the transcript from pC631Rluc. The inhibitory effect of IRNA on GLV-IRES-mediated translation of Rluc was abolished in the cells over-expressing GlLa (Fig. 4), thus reinforcing the conclusion that GlLa is required for GLV IRES function and that its inhibition by IRNA is through the interaction of IRNA with GlLa.

### An Over-expression of the C-terminal domain of GlLa Blocks the GlLa Function in *Giardia*

**Figure 5:**
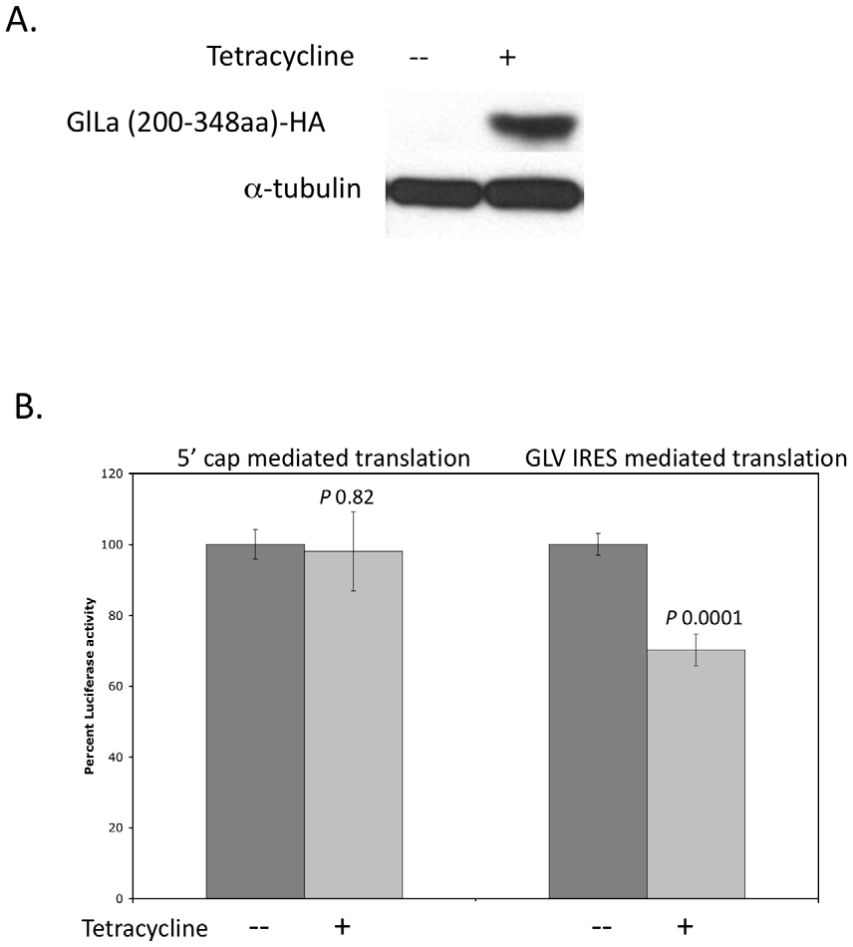
The dominant negative effect on GLV-IRES activity from over-expressing the C-terminal domain of GlLa. A) Western analysis with anti-HA antibody of un-induced and tetracycline induced *Giardia* cells over-expressing the HA-tagged C-terminal domain of GlLa. α-Tubulin was monitored as the loading control. B) Relative Rluc activities expressed from 5′-cap mediated or GLV-IRES mediated translation initiation in un-induced control (black) and tetracycline-induced (grey) GlLa C-terminal domain over-expression in *Giardia* trophozoites 3 hrs after transfection. The results were derived from three independent transfections (± S.D). *P* values are indicated above each bar in the graph.

Human La has been found to function as a dimer. An over-expression of the C-terminal domain of human La has an interfering effect on the dimerization and leads to inhibition of certain viral and cellular IRES mediated translation initiations *in vivo* [9]. In order to determine if GlLa also functions as a dimer and whether the dimerization could be inhibited by its C-terminal domain in *Giardia*, a 3XHA tagged GlLa C-terminal domain (aa 200–348) was over-expressed in *Giardia* using the Tet-inducible promoter system [30], and detected as a 21 KDa band in Western blot analysis after 24 hrs of Tet induction (Fig. 5A). The Tet-induced and un-induced cells were then transfected with the transcript from pC631Rluc and assayed for Rluc activity 5 hrs later. The Rluc activity was reduced by 30% in cells expressing the GlLa C-terminal domain when compared with the un-induced cells (Fig. 5B). In contrast, the cap-dependent translation of Rluc was unaffected by the GlLa C-terminal domain, indicating once again that GlLa is not involved in a cap-mediated translation (Fig. 5B). These results indicate that the C-terminal domain of GlLa has a dominant negative effect on GLV-IRES function, likely by inhibiting dimerization of GlLa.

### GlLa Localizes to the Cytoplasm of *Giardia* cells

**Figure 6:**
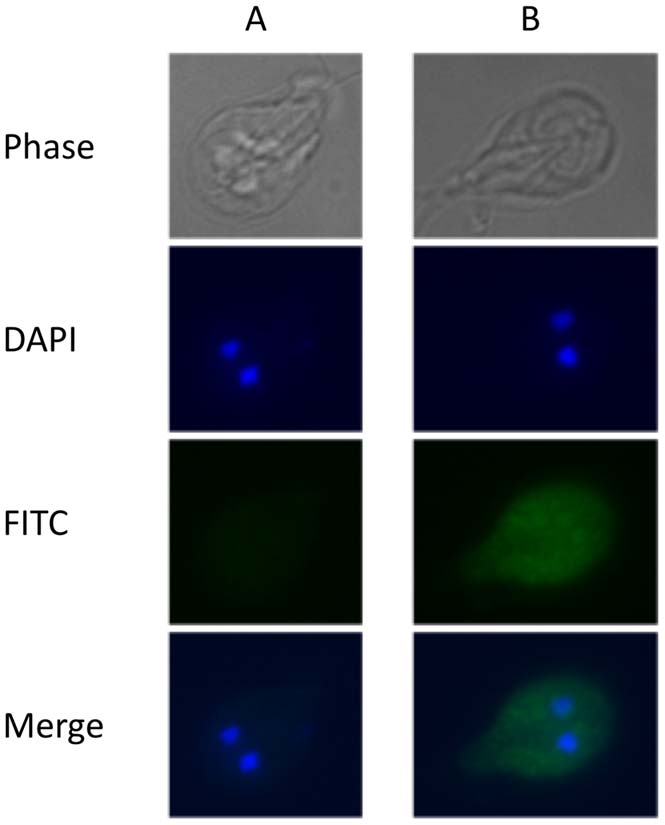
GlLa-HA localizes in the cytoplasm of *Giardia* trophozoites. Immunostaining of A) Wild type WB and B) GlLa-HA expressing WB *Giardia* cells with FITC labeled anti-HA antibody and visualized by Nikon TE2001 microscope.

La autoantigen is a nucleo-cytoplasmic protein and is primarily localized in the nucleus of mammalian and yeast cells [31]. To determine if GlLa also shares a similar localization pattern in *Giardia*, one of the four chromosomal copies of GlLa was tagged with 3XHA was generated using an endogenous tagging method as described previously [26]–[28]. When the cells expressing HA-tagged GlLa were immunostained with anti-HA antibodies and examined with fluorescence microscopy, the fluorescence was found primarily localized in the cytoplasm and very little was found in the nucleus (Fig. 6B), suggesting a primary role of GlLa in regulating GLV-IRES mediated translation, which takes place in the cytoplasm. Similar results were obtained when GFP tagged GlLa was used to observe its localization in the cell (data not shown).

## Discussion

In order to understand the mechanism of translation initiation at an IRES, it is essential to identify the cellular *trans*-acting proteins that bind to it and recruit ribosomes to initiate the process. Our current study demonstrates that GlLa is likely an essential auxiliary protein of GLV-IRES mediated translation. A partial depletion of endogenous GlLa protein from *Giardia* led to a corresponding decrease in GLV-IRES function, whereas an over-expression of GlLa stimulated it. GlLa binds to yeast IRNA and the latter competitively inhibits the binding of GlLa to radiolabeled GLV-IRES RNA *in vitro*. In *Giardia* trophozoites, introduction of yeast IRNA inhibited the function of GLV-IRES, but the inhibition could be reversed by an over-expression of GILa. All the evidence thus indicates that GlLa plays an essential role in GLV-IRES mediated translation in *Giardia*.

Extensive analysis has been done to understand the mechanism of human La function in IRES mediated translation. It was demonstrated that the N-terminal half of the protein containing the La motif, RRM1 and RRM 2, is involved in RNA binding [30]–[34], whereas the C-terminal domain is responsible for dimerization of the La protein [9]. It has been proposed that the dimer functions as a molecular chaperone by binding the secondary structures of various IRESs and inducing structural changes of the IRESs that favor further binding of other initiation factors and the small ribosome [9], [35], [36]. A dominant negative effect was observed in the C-terminal domain of La in preventing formation of the functional dimer [11]. Our current observation that an over-expressed C-terminal domain of GlLa has a dominant negative effect on the GLV-IRES function in *Giardia* suggests that GlLa may also first form a dimer via its C-terminal domain and then act on GLV-IRES [24].

Although little sequence similarity exists among IRESs, picornavirus IRESs do appear to share some similarities in their secondary structures and can be classified into three groups; enteroviruses and rhinoviruses (Type I IRES elements); cardioviruses and apthoviruses (Type II IRES elements); and hepatoviruses [37]. Free energy minimization modeling suggested that different types of IRESs could have a common three-dimensional structure core [38], providing the structural platform for bindings of ITAFs to different IRESs [39], [40]. GLV-IRES has a highly complex secondary structure [23], [24]. It does not belong to either Type I or II structure.

Human La is a nuclear protein of 408 amino acid residues shuttling between the cytoplasm and the nucleus [41], [42]. It is involved in a variety of cellular processes in addition to activating IRES mediated translation. It protects the 3′ ends of nascent pol III transcripts, ribosomal RNA processing and RNA transport across the nuclear membrane [43]–[45]. These features of La are effectively exploited by some of the viral as well as cellular IRESs. For poliovirus, the viral protease 3C_pro_ cleaves La at Gln358-Gly359 to remove the C-terminal nuclear localization signal of La and prevents its redistribution to the nucleus [46]. The truncated La, still retaining the C-terminal dimerization domain, is utilized for poliovirus IRES mediated translation in the cytoplasm [11], [35], [45]. Similarly, UV irradiation causes the redistribution of La into the cytoplasm where it stimulates IRES mediated translation of XIAP, a protein involved in preventing apoptosis [13]. In contrast, our study showed that GlLa localizes primarily in the cytoplasm of *Giardia* trophozoite. GlLa is a protein of 348 amino acid residues much smaller than human La and may not contain a corresponding nuclear targeting signal at the C-terminus [24].

Yeast IRNA has served as an invaluable tool in elucidating the essential role of human La protein in HCV and poliovirus IRES mediated translation initiation [15]–[17]. It was demonstrated that both sense and antisense sequences of IRNA fold into a similar secondary structure and exhibit similar binding affinities for La protein [15]. Thus, La may specifically recognize a secondary structure of IRNA rather than a specific nucleotide sequence in it. The same could also apply to the interaction between GlLa and IRNA. Since IRNA lacks any sequence similarity with GLV-IRES, the competition between the two in binding to GlLa may suggest similar secondary structure in the two RNA molecules recognizing and binding to GlLa. A related question would be whether *Giardia* has an IRNA-equivalent in interacting with GlLa and controlling the function of GLV-IRES. A BLAST of the *Giardia* genomic database with IRNA found two homologues. One (GL50803_6927) has a stretch of 26 bases matching 32 bases in IRNA (81% identity), whereas the other (GL50803_8865) has 31 bases identical to 44 bases in IRNA (70% identity) (unpublished work). While there is nothing known about the two homologues, further study will find out if they have transcripts functioning like yeast IRNA.

## Materials and Methods

### Endogenous Tagging of GlLa

Tagging one of the four copies of the endogenous GlLa gene was carried out [26] by following the original strategy developed for *Schizosaccharomyces pombe* [27], [28]. A portion of GlLa gene lacking the first encoded 30 amino acids was amplified by PCR and cloned in-frame with a C-terminal 3XHA tag into the endogenous tagging plasmid pc-3HABSR carrying a blasticidin (BSR) resistance marker [26]. The resulting construct pc-GlLa3HABSR was linearized at the Eco47III site located in the middle of the coding region of GlLa (Figure S1) and electroporated into *Giardia* WB strain (WB clone C6, ATCC 50803) trophozoites for homologous recombination between the 3′ half of the 3X-HA tagged GlLa gene and one of the endogenous GlLa genes [25], [26]. The transfected cells were selected for Blasticidin resistance (50 µg/mL of blasticidin) and maintained in 100 µg/mL of blasticidin for further analysis. The expression of the endogenously integrated 3XHA tagged GlLa was monitored by Western analysis using mouse anti-HA monoclonal antibody (Sigma).

### A Knockdown of GlLa Gene Expression with the Anti-sense Morpholinos

Expression of GlLa gene was post-transcriptionally inhibited using an anti-sense morpholino oligo essentially as previously described [25]. A 25mer morpholino-oligonucleotide complementary to the 9 nts of the 5′ UTR and 16 nts of the initial coding sequence of the GlLa mRNA was custom synthesized (Gene Tools, LLC). Approximately 30 µL of the 1 mM stock of either the antisense-GlLa morpholino-oligo or a standard control morpholino-oligo were electroporated into 3×10^6^ *Giardia* WB strain trophozoites expressing endogenously regulated 3XHA-tagged GlLa in 270 µL of the culture medium to generate a final morpholino-oligo concentration of 100 µM. The cells were incubated thereafter for 24, 48 and 72 hrs, respectively, and analyzed for reduction in the level of 3X-HA-GlLa protein with a Western analysis using the anti-HA antibody (Sigma).

### Gel Shift Assays

The cloning, expression and purification of full-length GlLa-6XHis fusion protein was described previously [24]. For gel shift assays, varying amounts of the fusion protein were mixed with approximately 100 ng of 5′-^32^P-labeled IRNA (see below) in the binding buffer (20 mM Tris, pH 7.6, 50 mM KCl, 2.5 mM MgOAc, 0.05% NP40, 1 mM DTT) and incubated at 30°C for 20 min. The RNA-protein complexes were fractionated in a 6% non-denaturing polyacrylamide gel and visualized by a phosophoimager (Amersham). For competition assays, RNA molecules with sequence of the multiple cloning site of pGEM-T plasmid was added as a non-specific competitor. For competition with the GLV-IRES RNA, the 5′UTR portion of GLV-IRES was synthesized and labeled with ^32^P and used for binding to the recombinant GlLa protein and competing with unlabeled IRNA or non-specific RNA.

### *In vitro* Transcription

The *in vitro* transcript of pC631Rluc was synthesized using Megascript T7 transcription kit as described previously [24]. The 5′-capped transcript of *Renilla* luciferase gene with a poly (A) tail was synthesized from the linearized pRL plasmid using a MessageMachine T7 transcription kit.

### *In vitro* Synthesis of IRNA

Two complementary oligonucleotide primers containing the *Saccharomyces cerevisiae* IRNA sequences [24] were annealed and inserted into the ApaI and EcoRI sites of pGEM-T easy vector to generate the pIRNA plasmid. IRNA transcript was generated from the linearized pIRNA plamsmid using T7 Megascript transcription kit and was purified using G-25 spin columns to remove un-incorporated nucleotides and enzymes. The IRNA thus synthesized was quantified using NanoDrop 2000 (Thermoscientific, USA).

### Transfection of *Giardia* Trophozoites and the Luciferase Assay

Transient transfection of *Giardia* WB strain trophozoites was performed as described previously [23]. The cells were harvested 5 hrs after transfection and the cell lysate was assayed for Rluc activities using *Renilla* Luciferase activity kit (Promega).

### Over-expression of 3XHA Tagged GlLa and GlLa C-terminal Domain in *Giardia*

Genes encoding full-length GlLa or the C-terminal domain (211–348aa) of GlLa were each amplified from *Giardia* genomic DNA with a modification of the stop codon and an incorporation of a NheI site at the 3′-end. The amplified sequence was cloned into the NcoI and EcoRI sites of pLop2 plasmid [30]. A sequence encoding 3XHA was inserted at the 3′-end of the full-length GlLa or the GlLa C-terminal domain using the NheI and EcoRI sites. The two fusion constructs were each moved into a pNLop2-GitetR plasmid using NheI and SalI sites as described previously [30]. The final constructs pNlop2-GlLaHA and pNlop2-LaDNHA were each electroporated into *Giardia* WB strain trophozoites and selected with 50 µg/mL of G418. The transfected cells were treated with 10 µg/mL of tetracycline for 24 hours to induce the expression of GlLa-3XHA or GlLa C-terminal domain-3XHA, which was monitored with Western analysis using the anti-HA antibody (Sigma). Anti-tubulin antibodies (Sigma) were used to monitor the loading controls.

### Immunoflourescence Assay

*Giardia* WB strain trophozoites expressing HA-tagged full-length GlLa at the endogenous level were harvested by placing the culture tubes on ice for 15 min, centrifuging them (2,500 rpm for 10 min.) to pellet the cells, suspending the cells in 1 ml of modified TYI-S- 33 culture medium, placing the suspension on cover slips pretreated with 0.1% poly-L-lysine, and incubating at 37°C for 30 min to allow the trophozoites to adhere. The attached cells were fixed with pre-warmed 4% paraformaldehyde for 30 min at room temperature and washed three times with PBS. The cells were then permeabilized with 0.5% Triton X-100 for 15 min at room temperature, washed three times with PBS, blocked with 5% BSA in TBS/TNT (20 mM Tris-HCl (pH 7.5), 150 mM NaCl, 0.3% Tween-20, 0.2% NP-40, and 0.05% Triton X-100) for 20 min and washed three times with TBS/TNT. The cells were then incubated with an Alexa Fluor 488-labeled anti-HA antibody (1∶500 in 0.1% BSA in TBS/TNT) for 60 min at room temperature and washed three times with TBS/TNT. The cover slip was then placed facedown on a clean glass slide with 1 drop of Vectashield mounting media with DAPI and sealed with clear nail polish. Cells were examined using a Nikon TE2000E motorized inverted microscope equipped with bright field and epifluorescence optics. Images were acquired with the NIS-Elements Advanced Research software and analyzed with ImageJ.

## Supporting Information

Figure S1 **Endogenous tagging of GlLa with a triple HA epitope.** GlLa gene lacking the first 100 nts (∼30aa) of the ORF is fused in frame at its 3′ end with the coding sequence of a triple HA epitope. The plasmid construct is linearized by Eco 47III at a unique restriction site located in the GlLa ORF and introduced into *Giardia* WB strain trophozoites by electroporation. Homologous recombination and integration of the linearized vector into the chromosomal copy of the GlLa gene generates a full length ORF with a triple HA tag at its 3′ end.
(TIF)

## References

[1] Merrick WC, Hershey JWB, Hershey JWB, Mathews MB, Sonenberg N (1996). The pathway and mechanism of eukaryotic protein synthesis.. translational control..

[2] Hellen CUT, Sarnow P (2001). Internal ribosome entry sites in eukaryotic mRNA molecules.. Genes and Dev.

[3] Stoneley M, Willis AE (2004). Cellular internal ribosome entry segments: structures, trans-acting factors and regulation of gene expression.. Oncogene.

[4] Baird SD, Turcotte M, Korneluk RG, Holcik M (2006). Searching for IRES.. RNA.

[5] Jang SK, Krausslich HG, Nicklin MJH, Duke GM, Palmenberg AC (1988). A segment of the 5′ nontranslated region of encephalomyocarditis virus RNA directs internal entry of ribosomes during *in vitro* translation.. J Virol.

[6] Martinez-Salas E, Pacheco A, Serrano P, Fernandez N (2008). New insights into internal ribosome entry site elements relevant from viral gene expression.. J Gen Virol.

[7] Spriggs KA, Bushell M, Mitchell SA, Willis AE (2005). Internal ribosome entry segment-mediated translation during apoptosis: the role of IRES-trans-acting factors.. Cell Death Differ.

[8] Meerowitch K, Pelletier J, Sonenberg N (1989). A cellular protein that binds to the 5′ noncoding region of poliovirus RNA: implications for internal translation initiation.. Genes Dev.

[9] Craig AW, Svitkin YV, Lee HS, Belsham GJ, Sonenberg N (1997). The La autoantigen contains a dimerization domain that is essential for enhancing translation.. Mol Cell Biol.

[10] Ali N, Siddiqui A (1997). The La autoantigen binds 5′ noncoding region of the hepatitis C virus in the context of the initiator AUG codon and stimulates internal ribosome entry site-mediated translation.. Proc Natl Acad Sci USA.

[11] Costa-Mattioli M, Svitkin Y, Sonenberg N (2004). La autoantigen is necessary for optimal function of the Poliovirus and Hepatitis C virus internal ribosome entry site *in vivo* and *in vitro*.. Mol Cell Biol.

[12] Kim YK, Jang SK (1999). La protein is required for efficient translation driven by encephalomyocarditis virus internal ribosomal entry site.. J Gen Virol.

[13] Holcik M, Korneluk RG (2000). Functional characterization of the X-linked inhibitor of apoptosis (XIAP) internal ribosome entry site element: role of La autoantigen in XIAP translation.. Mol Cell Biol.

[14] Kim YK, Back SH, Rho J, Lee SH, Jang SK (2001). La autoantigen enhances translation of BiP mRNA.. Nucl Acids Res.

[15] Venkatesan A, Das S, Dasgupta A (1999). Structure and function of a small RNA that selectively inhibits internal ribosome entry site-mediated translation.. Nucleic Acids Res.

[16] Das S, Coward P, Dasgutpa A (1994). A small yeast RNA selectively inhibits internal initiation of translation programmed by Poliovirus RNA: Specific interaction with cellular proteins that bind to the viral 5′-untranslated region.. J Virol.

[17] Das S, Kenan DJ, Bocskai D, Keene JD, Dasgupta A (1996). Sequences within a small yeast RNA required for inhibition of internal initiation of translation: interaction with La and other cellular proteins influences its inhibitory activity.. J Virol.

[18] Wang AL, Wang CC (1986). Discovery of a specific dougle-stranded RNA virus in Giardia lamblia.. Mol Biochem Parasitol.

[19] Yu DC, Wang AL, Wu CH, Wang CC (1995). Virus-mediated expression of firefly luciferase in the parasitic protozoan Giardia lamblia.. Mol Cell Biol.

[20] Garlapati S, Wang CC (2004). Identification of a novel internal ribosome entry site in giardiavirus that extends to both sides of the initiation codon.. J Biol Chem.

[21] Garlapati S, Chou J, Wang CC (2001). Specific secondary structures in capsid-coding region of Giardiavirus transcript are required for its translation in Giardia lamblia.. J Mol Biol.

[22] Garlapati S, Wang CC (2002). Identification of an essential pseudoknot in the putative downstream internal ribosome entry site in giardiavirus transcript.. RNA.

[23] Garlapati S, Wang CC (2005). Structural elements in the 5′untranslated region of Giardiavirus transcript essential for internal ribsome entry site-mediated translation initiation.. Eukary Cell.

[24] Garlapati S, Wang CC (2009). Giardiavirus internal ribosome entry site has an apparently unique mechanism of initiating translation.. PLoS one.

[25] Carpenter ML, Cande WZ (2009). Using morpholinos for gene knockdown in Giardia intestinalis.. Eukary Cell.

[26] Gourguechon S, Cande WZ (2011). Rapid tagging and integration of genes in Giardia intestinalis.. Eukary Cell.

[27] Forsburg SL, Sherman DA (1997). General purpose tagging vectors for fission yeast.. Gene.

[28] Craven SE, Griffiths DJ, Sheldrick KS, Randall RE, Hagan IM (1998). Vectors for the expression of tagged proteins in *Schizosaccharomyces pombe*.. Gene.

[29] Li L, Wang CC (2004). Capped mRNA with a single nucleotide leader is optimally translated in a primitive eukaryote Giardia lamblia.. J Biol Chem.

[30] Sun CH, Tai JH (2000). Development of a tetracycline controlled gene expression system in the parasitic protozoan *Giardia lamblia*.. Mol Biochem Parasitol.

[31] Bousquet-Antonelli C, Deragon JM (2009). A comprehensive analysis of the La-motif protein superfamily.. RNA.

[32] Izumi RE, Das S, Barat B, Ray CS, Dasgupta A (2004). A peptide from autoantigen La blocks Poliovirus and Hepatitis C virus cap-independent translation and reveals a single tyrosine critical for La RNA binding and translation stimulation.. J Virol.

[33] Dasgupta A, Das S, Izumi R, Venkatesan A, Barat B (2004). Targeting internal ribosome entry site (IRES)-mediated translation to block hepatitis C and RNA viruses.. FEMS Microbiol Lett.

[34] Pudi R, Ramamurthy SS, Das S (2005). A peptide derived from RNA recognition motif 2 of human La protein binds to hepatitis C virus internal ribosomal entry site, prevents ribosomal assembly, and inhibits internal initiation of translation.. J Virol.

[35] Svitkin YV, Meerovitch K, Lee HS, Dholakia JN, Kenan DJ (1994). Internal translation initiation on Poliovirus RNA: further characterization of La function in Poliovirus translation in vitro.. J Virol.

[36] Belsham GJ, Sonenberg N (2000). Picornavirus RNA translation: roles for cellular proteins.. Trends Microbiol.

[37] Ehrenfeld E, Hershey JWB, Mathews MB, Sonenberg N (1996). Initiation of translation by picornavirus RNAs.. Translational control.

[38] Le SY, Siddiqui A, Maizel JV (1996). A common structural core in the internal ribosome entry sites of picornavirus, hepatitis C virus and pestivirus.. Virus Genes.

[39] Niepmann M, Petersen A, Meyer K, Beck E (1997). Functional involvement of polypyrimidine tract-binding protein in translation initiation complexes with the internal ribosome entry site of foot-and-mouth disease virus.. J Virol.

[40] Gamarnik AV, Andino R (1997). Two functional complexes formed by KH domain containing proteins with the 5′ noncoding region of Poliovirus RNA.. RNA.

[41] Bachmann M, Pfeifer K, Schroder HC, Muller WE (1989). The La antigen shuttles between the nucleus and cytoplasm in CV-1 cells.. Mol Cell Biochem.

[42] Fok V, Friend K, Steitz JA (2006). Epstein-Barr virus noncoding RNAs are confined to the nucleus, whereas their partner, the human La protein, undergoes nucleocytoplasmic shuttling.. J Cell Biol.

[43] Wolin SL, Cedervall T (2002). The La protein.. Annu Rev Biochem.

[44] Maraia RJ, Intine RV (2002). La protein and its associated small nuclear and nucleolar precursor RNAs.. Gene Expr.

[45] Intine RV, Tenenbaum SA, Sakulich AL, Keene JD, Maraia RJ (2003). Differential phosphorylation and subcellular localization of La RNP's associated with precursor tRNAs and translation-related mRNAs.. Mol Cell.

[46] Bayfield MA, Yang R, Maraia RJ (2010). Conserved and divergent features of the structure and function of La and La-related proteins (LARPs).. Biochim Biophys Acta.

