# Supplementary figures and images for: A La Autoantigen Homologue Is Required for the Internal Ribosome Entry Site Mediated Translation of Giardiavirus
Source: PLoS One. 2011 Mar 29;6(3):e18263. doi: 10.1371/journal.pone.0018263 (PMC3066225; doi:10.1371/journal.pone.0018263)

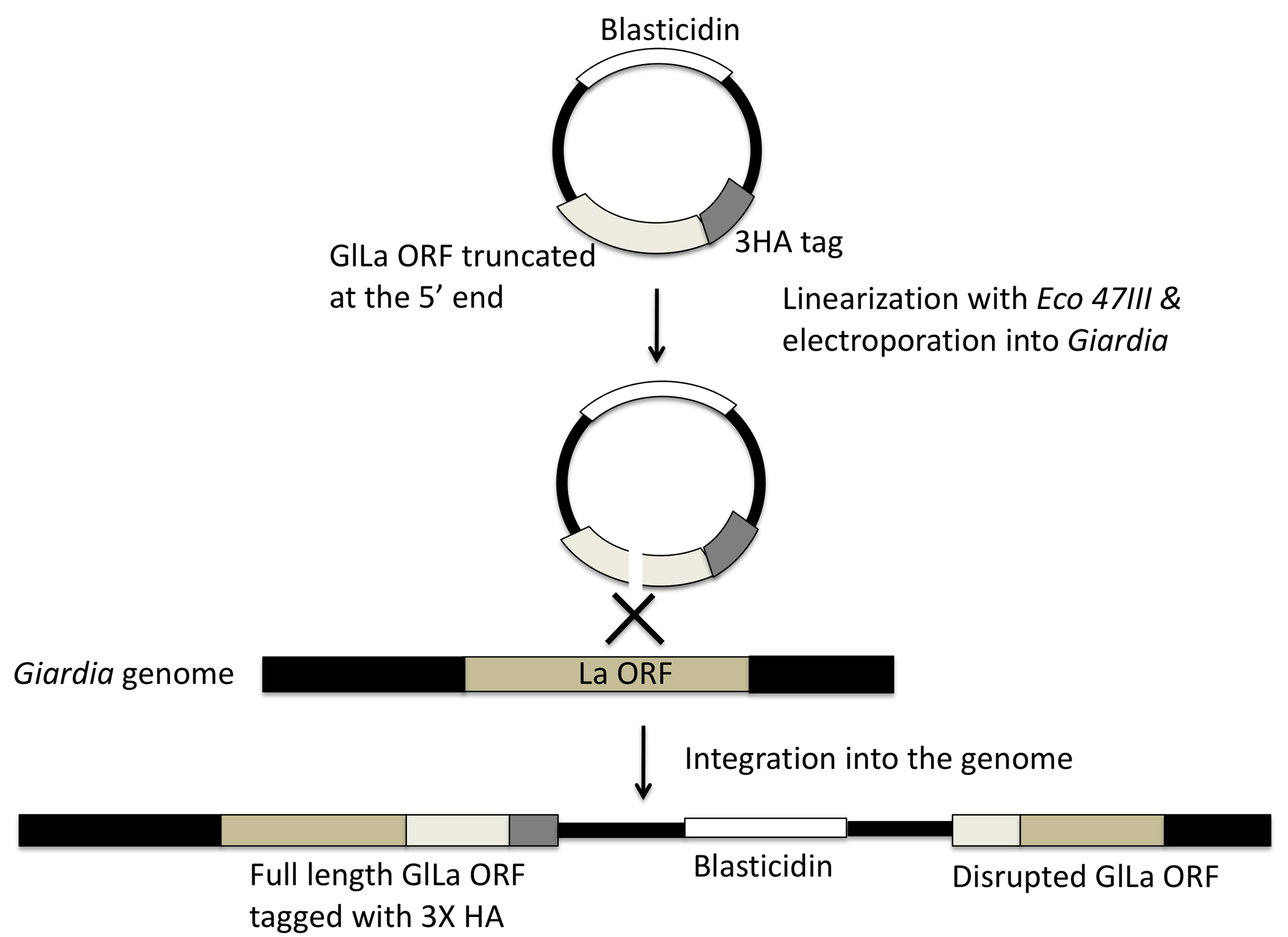

Supplement: Figure S1 — Endogenous tagging of GlLa with a triple HA epitope. GlLa gene lacking the first 100 nts (∼30aa) of the ORF is fused in frame at its 3′ end with the coding sequence of a triple HA epitope. The plasmid construct is linearized by Eco 47III at a unique restriction site located in the GlLa ORF and introduced into Giardia WB strain trophozoites by electroporation. Homologous recombination and integration of the linearized vector into the chromosomal copy of the GlLa gene generates a full length ORF with a triple HA tag at its 3′ end. (TIF) [file pone.0018263.s001.tif]
